# The Role and Regulation of Pulmonary Artery Smooth Muscle Cells in Pulmonary Hypertension

**DOI:** 10.1155/2020/1478291

**Published:** 2020-08-11

**Authors:** Shixin He, Tengteng Zhu, Zhenfei Fang

**Affiliations:** Department of Cardiology, The Second Xiangya Hospital of Central South University, No. 139, Middle Ren-Min Road, Changsha, Hunan 410011, China

## Abstract

Pulmonary hypertension (PH) is one of the most devastating cardiovascular diseases worldwide and it draws much attention from numerous scientists. As an indispensable part of pulmonary artery, smooth muscle cells are worthy of being carefully investigated. To elucidate the pathogenesis of PH, several theories focusing on pulmonary artery smooth muscle cells (PASMC), such as hyperproliferation, resistance to apoptosis, and cancer theory, have been proposed and widely studied. Here, we tried to summarize the studies, concentrating on the role of PASMC in the development of PH, feasible molecular basis to intervene, and potential treatment to PH.

## 1. Introduction

Pulmonary hypertension (PH) is a serious global health problem, which is characterized by progressing elevated pulmonary pressures and right heart failure, and mainly affects childbearing women [1]. The mean time from onset of symptoms to diagnosis is about 2 years, the mean survival time of idiopathic/heritable pulmonary arterial hypertension patients from treatment initiation is about 14.7 years, and the 10-year survival rates are 69.5% [2, 3]. Based on recent estimates, in the global population, the prevalence of PH is about 1%, while for individuals aged over 65 years, the number increases to 10%. What is more, about 80% of PH patients are living in developing countries [4].

The feature of PH is intense remodeling of small pulmonary arteries by myofibroblast and smooth muscle cell proliferation, and for familial pulmonary arterial hypertension, the bone morphogenetic protein type II receptor (BMPR-II) mutation in pulmonary artery smooth muscle cells contributes to abnormal growth responses to the transforming growth factor (TGF)-beta/bone morphogenetic protein (BMP) [5]. Compared to previous belief that vasoconstriction acts a vital role in PH pathogenesis [6, 7], there is a tendency to think that excessive proliferation and resistance to apoptosis of PASMC and pulmonary artery endothelial cells (PAEC) are the crucial components of pulmonary vascular remodeling [8]. PASMC has been widely proved to play an important role in the development of various types of pulmonary hypertension. Different mechanisms finally lead to uncontrolled proliferation of PASMC through apoptosis resistance, activated hypoxia-induced factor (HIF), HDAC modification, and inflammation, resulting in pulmonary hypertension [9, 10].

According to similar pathophysiological mechanisms, clinical presentation, haemodynamic characteristics, and therapeutic management, the clinical classification of PH is intended to categorize multiple clinical conditions into five groups [11]. Here, we mainly talk about WHO group 1 pulmonary arterial hypertension (PAH). To offer more suitable treatment and precisely evaluate patients' clinical outcome, the following parameters appear to have the greatest predictive capability: functional class, six-minute walk distance (6MWD), N-terminal pro-brain natriuretic peptide/brain natriuretic peptide (NT-proBNP/BNP) levels, cardiac index, right atrial pressure, and mixed venous oxygen saturation (SvO2) [12, 13]. Specific drug treatment of WHO group 1 PAH by targeting the nitric oxide, endothelin, and prostaglandin pathways has been the standard since 2003. Recently, based on different risk stratification, monotherapy or dual-combination therapies, including macitentan and sildenafil, riociguat and bosentan, selexipag and endothelin receptor antagonist (ERA) or phosphodiesterase inhibitor (PDE5i), or both, are recommended [14, 15].

## 2. Histopathology of Lungs in PH

### 2.1. Histology of Normal Lung Vessels

The major role of the right ventricle (RV) is to pump all the blood it receives per beat into the pulmonary circulation without elevating right atrial pressure. Normally, blood flow varies with minimum changes in pulmonary arterial pressure. Although the total compliance of the pulmonary circulation is about one-seventh that of the systemic circulation, it stores much less blood and has the ability to collapse pulmonary vessels as well as have them distended. Thus, the pulmonary circulation is able to accommodate increased blood volumes without increasing pulmonary artery pressure as much as would occur on the systemic circulation [16, 17].

### 2.2. Histopathology of PAH Lung Vessels

In 1958, Heath and Edwards [18] first described the histologic features of hypertensive pulmonary vascular structure changes into six grades in patients with congenital septal defects of the heart. The six grades included retention of fetal type pulmonary vessels, medial hypertrophy with cellular intimal reaction, progressive fibrous vascular occlusion, progressive generalized arterial dilatation with the formation of complex dilatation lesions (plexiform lesions), chronic dilatation with formation of numerous dilatation lesions and pulmonary hemosiderosis, and necrotizing arteritis. It is widely accepted that higher grade is related to worse pulmonary vessels and right heart function. Compared to the control groups, intima and intima plus media fractional thicknesses of pulmonary arteries were increased in the PAH group, in accordance with pulmonary haemodynamic measurements. There were remarkable perivascular inflammation in a mass of PAH lungs and correlated with intima plus media remodeling [19].

Pulmonary vasoconstriction caused by hypoxia was studied widely in PH [7]. As a result of global pulmonary hypoxic vasoconstriction, the right ventricular afterload could increase. Chronic hypoxia-induced PH is partly due to initial pulmonary artery contraction. Pulmonary artery pressures are higher in high-altitude dwellers with chronic mountain sickness, a syndrome including dyspnoea, fatigue, poor sleep, headache, and cyanosis. Hypoxic pulmonary vascular remodeling also contributes to PH and begins to develop within the first hours of hypoxic exposure. Hypoxia-induced PH in humans or animals is generally mild or moderate, but with a substantial afterload on the right ventricle during exercise. In vitro, hypoxia was reported to inhibit myocardial fibre contractility. Pulmonary vascular contraction plays an important role not only in hypoxic PH, but also in pulmonary arterial hypertension (PAH). Current pharmacological therapies for PAH mostly target pathways regulating endothelial factors with vasoconstrictive/vasodilatory and have made great achievements in improving the exercise capacity, haemodynamics, and time to clinical worsening of PAH patients.

It is increasingly believed that although vasospasm acts a role, pulmonary hypertension is an obstructive lung panvasculopathy and different forms of PH present with either a predominance of pulmonary arterial remodeling or vein remodeling or a variable contribution of both [20]. Obviously, there is medial and adventitial thickening of the pulmonary muscular and elastic vessels. The medial thickening is believed to result in hypertrophy and increased accumulation of smooth muscle cells as well as increased deposition of extracellular matrix proteins, predominantly collagen and elastin. The extent of structural changes, including SMC proliferation, hypertrophy, matrix protein production, and recruitment of adventitial or circulating cells, in the medial compartment of the pulmonary arterial wall partly determined the severity of chronic hypoxic pulmonary hypertension [21].

## 3. The Alteration of PASMC in PH

Data from post-mortem studies demonstrated medial hypertrophy, PASMC hyperproliferation, and muscle extension into distal arterioles, with important variability between individuals [22–25]. The accurate regulation of the balance between PASMC proliferation and apoptosis is significant in maintaining the normal integrity of structure and function in the pulmonary vessels. However, in severe angioproliferative PAH, this balance seems to be broken, following increased PASMC proliferation and decreased apoptosis, resulting in vessel wall thickening and vascular remodeling [26–31]. Contrast to previous belief that the relationship between pulmonary artery endothelial cells (PAEC) and PASMC is a simple one-way interaction from the endothelium to the PASMC, now it is more likely to believe that more complicated interactions exist between them [32–34]. Under abnormal or irritant conditions, the intricate interaction of PAEC and PASMC can be altered in the long term so that vascular proliferation and vasocontractility are enhanced further, which leads to PAH and right heart failure [35–38]. Owing to the characteristics of hyperproliferation and resistance to apoptosis of PASMC in PAH, there is an argument that PAH has something to do with cancer. At the molecular level, PASMC of PAH exhibits many features similar to cancer cells, which gives the chance to explore potential therapeutic treatments used in cancer to cure PAH [8, 39, 40].

## 4. Possible Pathways to Act on PASMC

### 4.1. Role of Ion Channels

It is well known that ions play many important roles in cell potential, cell contraction, and pH homeostasis, which can influence the proliferation and apoptosis of PASMC. Some studies demonstrated that decrease of K^+^ channels affected the PASMC depolarization, then facilitated vascular remodeling, and inhibited PASMC apoptosis. In PAH rat models, restoration of K^+^ channels activity and expression, using dehydroepiandrosterone or dichloroacetate, reduced pulmonary vascular remodeling. However, the exact mechanisms by which K^+^ channels act on PASMC are still controversial [41–51]. Lv et al. found increased expression of MicroRNA-206 suppressed potassium voltage-gated channel subfamily A member 5 (Kv1.5) and promoted the PASMC proliferation [52].

The elevated concentration of intracellular Ca^2+^ was found in PAH animal models and patients. This kind of phenomenon was not realized through activation of voltage-gated calcium channels (VGCC), but by increase of canonical transient receptor potential (TRPC) proteins, which involved Ca^2+^-permeable nonselective cation channels (NSCCs). Increased abundance of NSCCs was detected in PAH rat models and patients and inhibition of NSCCs, either pharmacologically or by RNA silencing, effectively decreased the concentration of intracellular Ca^2+^ and proliferation of PASMC [53–61]. Song et al. reported that stromal interaction molecule 2 (STIM2) protein, a Ca^2+^ sensor in the sarcoplasmic reticulum (SR) membrane, may contribute to elevated intracellular Ca^2+^ [62]. What is more, Ca^2+^ could activate nuclear factor of activated T-cells (NFAT), then suppress K^+^ channels expression, and lead to PASMC hyperproliferation [63]. It was also proved that hypoxia can cooperate with intracellular Ca^2+^, which increased the expression of aquaporin 1 (AQP1), a membrane water channel, indispensable for PASMC migration. Increased AQP1 upregulated *β*-catenin and its target genes (such as c-Myc and cyclin D1), which accelerated the proliferation and migration of PASMC [64–66].

The normal operation of Na^+^/H^+^ exchange (NHE) is essential to keep pH homeostasis of PASMC [67, 68]. Studies showed that increased expression of NHE isoform 1 (NHE1) can promote the exchange, elevate the pH, and induce the proliferation and migration of PASMC. Although the specific mechanisms are still unclear, it may have something to do with p27 (a cyclin-dependent kinase inhibitor), E2F1 (a nuclear transcription factor), and cytoskeletal re-arrangement [69–75].

### 4.2. Crucial Molecules

When we talk about PAH, we should never miss hypoxia and hypoxia-inducible factors (HIF). Under the circumstances of hypoxia, increased expression and decreased degradation result in accumulation of HIF-1*α*. A lot of studies proved that HIF-1*α* can influence the PASMC proliferation and mediate pulmonary vascular remodeling, by acting on Ca^2+^, pH homeostasis, endothelin-1 (ET-1), vascular endothelial growth factor (VEGF), and Warburg effect [76–84].

Endothelin is secreted by endothelial cells and has three isoforms, among which endothelin-1 (ET-1) is the most widely expressed and mediates vascular contraction, cell migration, and proliferation. In terms to PASMC, ET-1 binds to ET_A_ or ET_B_ and then has an impact on decreased K^+^ channels, elevated intracellular Ca^2+^, and activation of NHE1 and Rho kinase (ROCK) signaling, leading to the migration and proliferation of PASMC [85–88].

5-Hydroxytryptamine (5-HT) is well known in depression mechanism and it also takes part in the development of PAH. 5-HT enters PASMC through serotonin transporter (SERT). The signaling cascades caused by 5-HT include increased reactive oxygen species and activation of mitogen-activated protein kinase (MAPK) and ROCK pathway, which regulate the expression of genes targeting cell growth and influence PASMC [89–93].

### 4.3. Important Pathways

#### 4.3.1. Rho Kinase

Rho kinase (ROCK) signaling pathway plays an indispensable part in vascular contraction and remodeling. Exposed to hypoxia, activation of ROCK in PASMC through Rho B (upstream activators of ROCK) could augment the proliferation and migration of PASMC, resulting in increased pulmonary vascular resistance. There were studies stating that long-term use of ROCK inhibitors could ameliorate vascular remodeling [94–103]. Abe et al. reported that PDGF activated ROCK, suppressed the translocation of Smad1 originally induced by bone morphogenetic protein 2 (BMP 2), and increased PASMC proliferation [104].

#### 4.3.2. BMP Signaling

**Figure 1:**
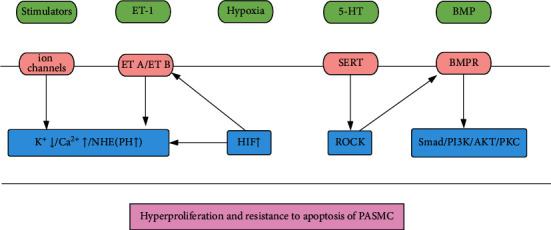
Molecular pathways in PASMC (1). ET-1: endothelin-1, 5-HT: serotonin, BMP: bone morphogenetic proteins, ET A/ET B endothelin receptor A/B, SERT: serotonin transporter, BMPR: bone morphogenetic proteins receptor, NHE: Na^+^/H^+^ exchanger, HIF: hypoxia-induced factor, ROCK: Rho kinase, PI3K/AKT: phosphatidylinositide 3-kinase/protein kinase B, PKC: protein kinase C.

Bone morphogenetic protein receptor type 2 (BMPR2) mutations are present in patients with heritable and idiopathic PAH, which reminds us of BMP signaling's significant role in the development of PAH. The mutation of BMPR2 could inhibit the antiproliferation effect of BMP2, leading to PAH. BMP can exert its function in a way of Smad dependent or independent. BMP/BMPR1 interacts with Smad1/5/8, then increasing their binding with Smad4, finally leading to elevated related genes expression. In other ways, BMP activates MAPK, PI3K/AKT, or protein kinase C (PKC) to influence PASMC. The impaired control of BMP signaling may be a common characteristic of PH no matter what the pathogenesis is [105–110] (Figure 1).

#### 4.3.3. Cancer Theories

As mentioned above, at the molecular level, PASMC of PAH exhibits many features similar to cancer cells, making it possible to explore potential therapeutic treatments used in cancer to cure PAH (reviewed in [40]). Studies showed increase of IL-6, monocyte chemotactic protein 1 (MCP-1), and tumor necrosis factor alpha (TNF-*α*) related to worse clinical outcomes in PAH patients. IL-6 knockout effectively ameliorated PAH in animal models. Platelet-derived growth factor (PDGF) mediated mitogenic signaling and thickening of the pulmonary vascular media. These growth factors and inflammatory mediators eventually have an impact on cell growth and survival by MEK/ERK, PI3K/AKT, or JAK/STAT3 pathways. In PASMC, it was reported that activation of STAT3 can upregulate the expression of proviral integration site for Moloney murine leukemia virus-1 (PIM-1) and then enhance NFAT-mediated transactivation, resulting in decreased K^+^ channels and increased intracellular Ca^2+^. In addition, activation of PI3K/AKT and JAK/STAT3 inhibited the transcription factor Forkhead box protein O1 (FOXO1), causing elevated Cyclin B1 and D1 and decreased p27, which promoted PASMC proliferation [48, 111–122].

Mammalian target of rapamycin (mTOR) signaling plays important roles in cell metabolism, cell proliferation, and survival. Together with other proteins, mTOR forms two independent complexes, mTORC1 (mTOR-Raptor) and mTORC2 (mTOR-Rictor). Activation of mTORC1 could enhance ribosomal protein S6 kinase beta-1 (S6K1) and suppress eukaryotic translation initiation factor 4E-binding protein 1 (4E-BP1), which facilitates cell growth and proliferation. On the other hand, mTORC2 is more likely to respond to growth factors, increasing cell survival [123–125]. However, Tang et al. reported that mTORC1 and mTORC2 had different roles in the development of PAH. Inhibition of mTORC1 ameliorated pulmonary hypertension, while inhibition of mTORC2 facilitated spontaneous pulmonary hypertension and it may result from upregulation of PDGF receptors in PASMC [126].

The Hippo signaling pathway is believed to relate to controlling organ size. It is constitutive of a cascade of tumor suppressive kinases mammalian STE20-like protein kinase 1/2 (MST1/2) and large tumor suppressor homolog 1/2 (LATS1/2), while its downstream molecules include yes-associated protein 1 (YAP) and transcriptional coactivator with PDZ-binding motif (TAZ). Inactivation of LATS1/2 leads to decrease of YAP and TAZ in cytoplasm and activation of HIF-1*α* and Notch3 pathways, which plays a deleterious role in the development of PAH [127–133].

Most cancer cells rely on aerobic glycolysis, instead of depending on mitochondrial oxidative phosphorylation to generate energy, a phenomenon termed “the Warburg effect.” This effect also can be seen in PASMC and PAH. Driven by HIF activation, augmented glycolysis is characterized by elevated expression of pivotal proteins in its pathway, such as glucose transporters, hexokinase, pyruvate dehydrogenase kinase (PDK), lactate dehydrogenase (LDH), and 6-phosphofructo-2-kinase/fructose-2,6-bisphosphatase 3 (PFKFB3). By interacting with PI3K/AKT, ERK1/2, and HIF-1*α* and altering the morphology and subcellular distribution of mitochondria, Warburg effect increases the proliferation of PASMC in PAH [10, 134–144].

#### 4.3.4. Other Pathways

**Figure 2:**
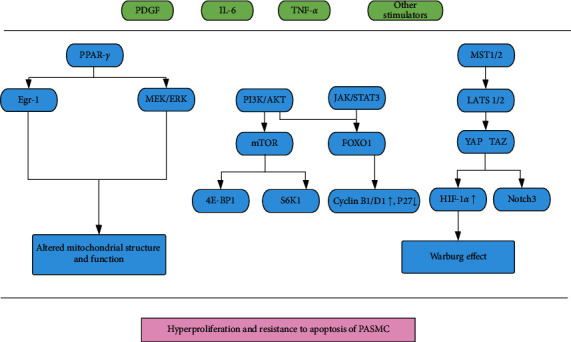
Molecular pathways in PASMC (2). PDGF: platelet-derived growth factor, IL-6: interleukin-6, TNF-*α*: tumor necrosis factor-*α*, PPAR-*γ*: peroxisome proliferator-activated receptor-*γ*, MST 1/2: mammalian sterile 20-like kinases 1/2, Egr-1: early growth response-1, MEK/ERK: mitogen-activated protein kinase/extracellular-signal regulated kinase, PI3K/AKT: phosphatidylinositide 3-kinase/protein kinase B, JAK/STAT3: Janus kinase/signal transducer and activator of transcription 3, LATS 1/2: large tumor suppressor kinases 1/2, mTOR: mechanistic target of rapamycin, FOXO1: forkhead box protein O1, YAP: yes-associated protein, TAZ: transcriptional coactivator with PDZ-binding motif, 4E-BP1: eukaryotic translation initiation factor 4E-binding protein 1, S6K1: ribosomal protein S6 kinase beta-1, HIF-1*α*: hypoxia-induced factor-1*α*.

Peroxisome proliferator-activated receptor *γ* (PPAR*γ*) regulates mitochondrial gene expression and biogenesis. Loss of PPAR*γ* leads to derangement in mitochondrial structure and function, which has a harmful impact on PASMC and PAH [145]. Xie et al. stated that leptin effectively ameliorated pulmonary vascular remodeling and PAH, via activation of ERK1/2 and elevated expression of early growth response-1 (Egr-1), resulting in loss of PPAR*γ* [146]. In addition, Li et al. reported that activating prostanoid EP4 receptor (EP4) also decreased the expression of PPAR*γ* through protein kinase A (PKA) pathway and attenuated pulmonary arterial remodeling [147] (Figure 2).

Cyclin-dependent kinases (CDK) are crucial regulators of cell cycle and proliferation. Dinaciclib and palbociclib inhibited specific CDK and decreased PASMC proliferation via cell cycle arrest and interacted with the downstream CDK-Rb (retinoblastoma protein)-E2F signaling pathway, offering a potential strategy in PAH [148]. Sphingosine kinase 1 (SphK1) is a lipid kinase for phosphorylating sphingosine to generate sphingosine-1-phosphate (S1P). SphK1/S1P have been reported to relate to cell proliferation, migration, and survival. TGF-*β*1 could phosphorylate Smad2/3 and then elevate the expression of SphK1 and S1P, which activates Notch3 pathway to promote PASMC proliferation [149]. What is more, Sysol et al. reported that decreased micro-RNA-1 induced by hypoxia had an effect on the development of PAH via regulation of sphingosine kinase 1 [150].

## 5. Potential Treatment to PAH

While calcium channel blockers, endothelin receptor antagonists, phosphodiesterase type 5 inhibitors and guanylate cyclase stimulators, prostacyclin analogues, and prostacyclin receptor agonists are the classical specific drug therapies for PAH, their effects still are limited and unsatisfactory. Based on the molecular pathways mentioned above, tyrosine kinase inhibitors (platelet-derived growth factor inhibitors) and serotonin antagonists are being explored, but present outcomes are not ideal. Moreover, ROCK inhibitors, VEGF receptor inhibitors, stem cell therapy, mTOR inhibitors, PPAR-*γ* agonist, and strategies aiming at Warburg effect are all in the early phase of research [15, 142–144, 151, 152].

## 6. Summary

Although the treatment for pulmonary hypertension has achieved great improvement, it is still not that satisfactory. Owing to its indispensable role in the development of pulmonary hypertension, PASMC becomes the research hot spot in PH. Further elucidating the molecular basis of PASMC, including ion channels, HIF, ET-1, ROCK, BMP, PPAR-*γ*, and Warburg effect, could bring hope to PH treatment.

## References

[1] McLaughlin V. V., Archer S. L., Badesch D. B. (2009). ACCF/AHA 2009 expert consensus document on pulmonary hypertension. *Journal of the American College of Cardiology*.

[2] Rich S., Dantzker D. R., Ayres S. M. (1987). Primary pulmonary hypertension. A national prospective study. *Annals of Internal Medicine*.

[3] Ogawa A., Satoh T., Tamura Y., Fukuda K., Matsubara H. (2017). Survival of Japanese patients with idiopathic/heritable pulmonary arterial hypertension. *American Journal of Cardiology*.

[4] Hoeper M. M., Humbert M., Souza R. (2016). A global view of pulmonary hypertension. *The Lancet Respiratory Medicine*.

[5] Morrell N. W. (2006). Pulmonary hypertension due to BMPR2 mutation: a new paradigm for tissue remodeling?. *Proceedings of the American Thoracic Society*.

[6] Motley H. L., Cournand A., Werko L., Himmelstein A., Dresdaleand D. (1947). The influence of short periods of induced acute anoxia upon pulmonary artery pressures in man. *American Journal of Physiology-Legacy Content*.

[7] Naeije R., Dedobbeleer C. (2013). Pulmonary hypertension and the right ventricle in hypoxia. *Experimental Physiology*.

[8] Boucherat O., Vitry G., Trinh I., Paulin R., Provencher S., Bonnet S. (2017). The cancer theory of pulmonary arterial hypertension. *Pulmonary Circulation*.

[9] Perez V. A. D. J. (2016). Molecular pathogenesis and current pathology of pulmonary hypertension. *Heart Failure Reviews*.

[10] Paulin R., Michelakis E. D. (2014). The metabolic theory of pulmonary arterial hypertension. *Circulation Research*.

[11] Simonneau G., Montani D., Celermajer D. S. (2019). Haemodynamic definitions and updated clinical classification of pulmonary hypertension. *European Respiratory Journal*.

[12] Sitbon O., Benza R. L., Badesch D. B. (2015). Validation of two predictive models for survival in pulmonary arterial hypertension. *European Respiratory Journal*.

[13] Boucly A., Weatherald J., Humbert M., Sitbon O. (2018). Risk assessment in pulmonary arterial hypertension. *European Respiratory Journal*.

[14] Condon D. F., Nickel N. P., Anderson R., Mirza S., Perez V. A. D. J. (2019). The 6th world symposium on pulmonary hypertension: what’s old is new. *F1000Research*.

[15] Galie N., Humbert M., Vachiery J.-L. (2016). 2015 ESC/ERS guidelines for the diagnosis and treatment of pulmonary hypertension: the joint task force for the diagnosis and treatment of pulmonary hypertension of the European society of cardiology (ESC) and the European respiratory society (ERS): endorsed by: association for European paediatric and congenital cardiology (AEPC), international society for heart and lung transplantation (ISHLT). *European Heart Journal*.

[16] Pinsky M. R. (2016). The right ventricle: interaction with the pulmonary circulation. *Critical Care*.

[17] He Y.-Y., Liu C.-L., Li X., Li R.-J., Wang L.-L., He K.-L. (2016). Salubrinal attenuates right ventricular hypertrophy and dysfunction in hypoxic pulmonary hypertension of rats. *Vascular Pharmacology*.

[18] Heath D., Edwards J. E. (1958). The pathology of hypertensive pulmonary vascular disease; a description of six grades of structural changes in the pulmonary arteries with special reference to congenital cardiac septal defects. *Circulation*.

[19] Stacher E., Graham B. B., Hunt J. M. (2012). Modern age pathology of pulmonary arterial hypertension. *American Journal of Respiratory and Critical Care Medicine*.

[20] Tuder R. M., Archer S. L., Dorfmuller P. (2013). Relevant issues in the pathology and pathobiology of pulmonary hypertension. *Journal of the American College of Cardiology*.

[21] Stenmark K. R., Fagan K. A., Frid M. G. (2006). Hypoxia-induced pulmonary vascular remodeling: cellular and molecular mechanisms. *Circulation Research*.

[22] Naeye R. L. (1961). Hypoxemia and pulmonary hypertension. A study of the pulmonary vasculature. *Archives of Pathology*.

[23] Naeye R. L. (1965). Children at high altitude: pulmonary and renal abnormalities. *Circulation Research*.

[24] Arias-Stella J., Saldana M. (1963). The terminal portion of the pulmonary arterial tree in people native to high altitudes. *Circulation*.

[25] Heath D., Smith P., Rios D. J., Williams D., Harris P. (1981). Small pulmonary arteries in some natives of La Paz, Bolivia. *Thorax*.

[26] Rabinovitch M. (1998). Elastase and the pathobiology of unexplained pulmonary hypertension. *Chest*.

[27] Rubin L. J. (1999). Cellular and molecular mechanisms responsible for the pathogenesis of primary pulmonary hypertension. *Pediatric Pulmonology*.

[28] Wagenvoort C. A. (1970). The pathology of primary pulmonary hypertension. *Journal of Pathology*.

[29] Wohrley J. D., Frid M. G., Moiseeva E. P., Orton E. C., Belknap J. K., Stenmark K. R. (1995). Hypoxia selectively induces proliferation in a specific subpopulation of smooth muscle cells in the bovine neonatal pulmonary arterial media. *Journal of Clinical Investigation*.

[30] Diez J., Fortuno M. A., Zalba G. (1998). Altered regulation of smooth muscle cell proliferation and apoptosis in small arteries of spontaneously hypertensive rats. *European Heart Journal*.

[31] Sakao S., Tatsumi K., Voelkel N. F. (2009). Endothelial cells and pulmonary arterial hypertension: apoptosis, proliferation, interaction and transdifferentiation. *Respiratory Research*.

[32] Balcells M., Martorell J., Olive C. (2010). Smooth muscle cells orchestrate the endothelial cell response to flow and injury. *Circulation*.

[33] Billaud M., Lohman A. W., Johnstone S. R., Biwer L. A., Mutchler S., Isakson B. E. (2014). Regulation of cellular communication by signaling microdomains in the blood vessel wall. *Pharmacological Reviews*.

[34] Nogueira-Ferreira R., Ferreira R., Henriques-Coelho T. (2014). Cellular interplay in pulmonary arterial hypertension: implications for new therapies. *Biochimica et Biophysica Acta (BBA)-Molecular Cell Research*.

[35] Ivy D. D., Abman S. H., Barst R. J. (2013). Pediatric pulmonary hypertension. *Journal of the American College of Cardiology*.

[36] Austin E. D., Kawut S. M., Gladwin M. T., Abman S. H. (2014). Pulmonary hypertension: NHLBI workshop on the primary prevention of chronic lung diseases. *Annals of the American Thoracic Society*.

[37] Guignabert C., Tu L., Girerd B. (2015). New molecular targets of pulmonary vascular remodeling in pulmonary arterial hypertension: importance of endothelial communication. *Chest*.

[38] Gao Y., Chen T., Raj J. U. (2016). Endothelial and smooth muscle cell interactions in the pathobiology of pulmonary hypertension. *American Journal of Respiratory Cell and Molecular Biology*.

[39] Voelkel N. F., Cool C., Lee S. D., Wright L., Geraci M. W., Tuder R. M. (1998). Primary pulmonary hypertension between inflammation and cancer. *Chest*.

[40] Guignabert C., Tu L., Hiress M. L. (2013). Pathogenesis of pulmonary arterial hypertension: lessons from cancer. *European Respiratory Review*.

[41] Suzuki H., Twarog B. M. (1982). Membrane properties of smooth muscle cells in pulmonary hypertensive rats. *American Journal of Physiology-Heart and Circulatory Physiology*.

[42] Shimoda L. A., Polak J. (2011). Hypoxia. 4. Hypoxia and ion channel function. *American Journal of Physiology-Cell Physiology*.

[43] Yuan J. X., Aldinger A. M., Juhaszova M. (1998). Dysfunctional voltage-gated K^+^ channels in pulmonary artery smooth muscle cells of patients with primary pulmonary hypertension. *Circulation*.

[44] Burg E. D., Remillard C. V., Yuan J. X.-J. (2008). Potassium channels in the regulation of pulmonary artery smooth muscle cell proliferation and apoptosis: pharmacotherapeutic implications. *British Journal of Pharmacology*.

[45] Michelakis E. D., McMurtry M. S., Wu X.-C. (2002). Dichloroacetate, a metabolic modulator, prevents and reverses chronic hypoxic pulmonary hypertension in rats: role of increased expression and activity of voltage-gated potassium channels. *Circulation*.

[46] Bonnet S., Michelakis E. D., Porter C. J. (2006). An abnormal mitochondrial-hypoxia inducible factor-1*α*-Kv channel pathway disrupts oxygen sensing and triggers pulmonary arterial hypertension in fawn hooded rats: similarities to human pulmonary arterial hypertension. *Circulation*.

[47] Yuan X.-J., Wang J., Juhaszova M., Gaine S. P., Rubin L. J. (1998). Attenuated K^+^ channel gene transcription in primary pulmonary hypertension. *The Lancet*.

[48] Bonnet S., Rochefort G., Sutendra G. (2007). The nuclear factor of activated T cells in pulmonary arterial hypertension can be therapeutically targeted. *Proceedings of the National Academy of Sciences*.

[49] Pozeg Z. I., Michelakis E. D., McMurtry M. S. (2003). In vivo gene transfer of the O2-sensitive potassium channel Kv1.5 reduces pulmonary hypertension and restores hypoxic pulmonary vasoconstriction in chronically hypoxic rats. *Circulation*.

[50] Bonnet S., Dumas-De-La-Roque E., Begueret H. (2003). Dehydroepiandrosterone (DHEA) prevents and reverses chronic hypoxic pulmonary hypertension. *Proceedings of the National Academy of Sciences*.

[51] McMurtry M. S., Bonnet S., Wu X. (2004). Dichloroacetate prevents and reverses pulmonary hypertension by inducing pulmonary artery smooth muscle cell apoptosis. *Circulation Research*.

[52] Lv Y., Fu L., Zhang Z. (2019). Increased expression of MicroRNA-206 inhibits potassium voltage-gated channel subfamily A member 5 in pulmonary arterial smooth muscle cells and is related to exaggerated pulmonary artery hypertension following intrauterine growth retardation in rats. *Journal of the American Heart Association*.

[53] Wang J., Weigand L., Lu W., Sylvester J. T., Semenza G. L., Shimoda L. A. (2006). Hypoxia inducible factor 1 mediates hypoxia-induced TRPC expression and elevated intracellular Ca^2+^ in pulmonary arterial smooth muscle cells. *Circulation Research*.

[54] Lin M.-J., Leung G. P. H., Zhang W.-M. (2004). Chronic hypoxia-induced upregulation of store-operated and receptor-operated Ca^2+^ channels in pulmonary arterial smooth muscle cells: a novel mechanism of hypoxic pulmonary hypertension. *Circulation Research*.

[55] Golovina V. A., Platoshyn O., Bailey C. L. (2001). Upregulated TRP and enhanced capacitative Ca^2+^ entry in human pulmonary artery myocytes during proliferation. *American Journal of Physiology-Heart and Circulatory Physiology*.

[56] Landsberg J. W., Yuan J. X.-J. (2004). Calcium and TRP channels in pulmonary vascular smooth muscle cell proliferation. *Physiology*.

[57] Leggett K., Maylor J., Undem C. (2012). Hypoxia-induced migration in pulmonary arterial smooth muscle cells requires calcium-dependent upregulation of aquaporin 1. *American Journal of Physiology-Lung Cellular and Molecular Physiology*.

[58] Liu X.-R., Zhang M.-F., Yang N. (2012). Enhanced store-operated Ca^2+^ entry and TRPC channel expression in pulmonary arteries of monocrotaline-induced pulmonary hypertensive rats. *American Journal of Physiology-Cell Physiology*.

[59] Song M. Y., Makino A., Yuan J. X.-J. (2011). STIM2 contributes to enhanced store-operated Ca entry in pulmonary artery smooth muscle cells from patients with idiopathic pulmonary arterial hypertension. *Pulmonary Circulation*.

[60] Yu Y., Fantozzi I., Remillard C. V. (2004). Enhanced expression of transient receptor potential channels in idiopathic pulmonary arterial hypertension. *Proceedings of the National Academy of Sciences*.

[61] Kunichika N., Landsberg J. W., Yu Y. (2004). Bosentan inhibits transient receptor potential channel expression in pulmonary vascular myocytes. *American Journal of Respiratory and Critical Care Medicine*.

[62] Song S., Carr S. G., McDermott K. M. (2018). STIM2 (stromal interaction molecule 2)-mediated increase in resting cytosolic free Ca^2+^ concentration stimulates PASMC proliferation in pulmonary arterial hypertension. *Hypertension*.

[63] He R.-L., Wu Z.-J., Liu X.-R., Gui L.-X., Wang R.-X., Lin M.-J. (2018). Calcineurin/NFAT signaling modulates pulmonary artery smooth muscle cell proliferation, migration and apoptosis in monocrotaline-induced pulmonary arterial hypertension rats. *Cellular Physiology and Biochemistry*.

[64] Saadoun S., Papadopoulos M. C., Hara-Chikuma M., Verkman A. S. (2005). Impairment of angiogenesis and cell migration by targeted aquaporin-1 gene disruption. *Nature*.

[65] Monzani E., Bazzotti R., Perego C., Porta C. A. M. L. (2009). AQP1 is not only a water channel: it contributes to cell migration through Lin7/beta-catenin. *PLoS One*.

[66] Yun X., Jiang H., Lai N., Wang J., Shimoda L. A. (2017). Aquaporin 1-mediated changes in pulmonary arterial smooth muscle cell migration and proliferation involve beta-catenin. *American Journal of Physiology-Lung Cellular and Molecular Physiology*.

[67] Madden J. A., Ray D. E., Keller P. A., Kleinman J. G. (2001). Ion exchange activity in pulmonary artery smooth muscle cells: the response to hypoxia. *American Journal of Physiology-Lung Cellular and Molecular Physiology*.

[68] Quinn D. A., Honeyman T. W., Joseph P. M., Thompson B. T., Hales C. A., Scheid C. R. (1991). Contribution of Na^+^/H^+^ exchange to pH regulation in pulmonary artery smooth muscle cells. *American Journal of Respiratory Cell and Molecular Biology*.

[69] Quinn D. A., Dahlberg C. G., Bonventre J. P. (1996). The role of Na^+^/H^+^ exchange and growth factors in pulmonary artery smooth muscle cell proliferation. *American Journal of Respiratory Cell and Molecular Biology*.

[70] Rios E. J., Fallon M., Wang J., Shimoda L. A. (2005). Chronic hypoxia elevates intracellular pH and activates Na^+^/H^+^ exchange in pulmonary arterial smooth muscle cells. *American Journal of Physiology-Lung Cellular and Molecular Physiology*.

[71] Shimoda L. A., Fallon M., Pisarcik S., Wang J., Semenza G. L. (2006). HIF-1 regulates hypoxic induction of NHE1 expression and alkalinization of intracellular pH in pulmonary arterial myocytes. *American Journal of Physiology-Lung Cellular and Molecular Physiology*.

[72] Quinn D. A., Du H.-K., Thompson B. T., Hales C. A. (1998). Amiloride analogs inhibit chronic hypoxic pulmonary hypertension. *American Journal of Respiratory and Critical Care Medicine*.

[73] Yu L., Quinn D. A., Garg H. G., Hales C. A. (2008). Deficiency of the NHE1 gene prevents hypoxia-induced pulmonary hypertension and vascular remodeling. *American Journal of Respiratory and Critical Care Medicine*.

[74] Yu L., Hales C. A. (2011). Silencing of sodium-hydrogen exchanger 1 attenuates the proliferation, hypertrophy, and migration of pulmonary artery smooth muscle cells via E2F1. *American Journal of Respiratory Cell and Molecular Biology*.

[75] Denker S. P., Huang D. C., Orlowski J., Furthmayr H., Barber D. L. (2000). Direct binding of the Na-H exchanger NHE1 to ERM proteins regulates the cortical cytoskeleton and cell shape independently of H^+^ translocation. *Molecular Cell*.

[76] Semenza G. L. (2005). Pulmonary vascular responses to chronic hypoxia mediated by hypoxia-inducible factor 1. *Proceedings of the American Thoracic Society*.

[77] Prabhakar N. R., Semenza G. L. (2012). Adaptive and maladaptive cardiorespiratory responses to continuous and intermittent hypoxia mediated by hypoxia-inducible factors 1 and 2. *Physiological Reviews*.

[78] Yu A. Y., Shimoda L. A., Iyer N. V. (1999). Impaired physiological responses to chronic hypoxia in mice partially deficient for hypoxia-inducible factor 1alpha. *Journal of Clinical Investigation*.

[79] Shimoda L. A., Semenza G. L. (2011). HIF and the lung: role of hypoxia-inducible factors in pulmonary development and disease. *American Journal of Respiratory and Critical Care Medicine*.

[80] Shimoda L. A. (2012). 55th Bowditch Lecture: effects of chronic hypoxia on the pulmonary circulation: role of HIF-1. *Journal of Applied Physiology*.

[81] Iyer N. V., Kotch L. E., Agani F. (1998). Cellular and developmental control of O2 homeostasis by hypoxia-inducible factor 1 alpha. *Genes & Development*.

[82] Shimoda L. A. (2010). Hypoxic regulation of ion channels and transporters in pulmonary vascular smooth muscle. *Advances in Experimental Medicine and Biology*.

[83] Wang C.-C., Ying L., Barnes E. A. (2018). Pulmonary artery smooth muscle cell HIF-1alpha regulates endothelin expression via microRNA-543. *American Journal of Physiology-Lung Cellular and Molecular Physiology*.

[84] He X., Song S., Ayon R. J. (2018). Hypoxia selectively upregulates cation channels and increases cytosolic [Ca^2+^] in pulmonary, but not coronary, arterial smooth muscle cells. *American Journal of Physiology-Cell Physiology*.

[85] Shao D., Park J. E. S., Wort S. J. (2011). The role of endothelin-1 in the pathogenesis of pulmonary arterial hypertension. *Pharmacological Research*.

[86] Shimoda L. A., Sham J. S. K., Liu Q., Sylvester J. T. (2002). Acute and chronic hypoxic pulmonary vasoconstriction: a central role for endothelin-1?. *Respiratory Physiology & Neurobiology*.

[87] Whitman E. M., Pisarcik S., Luke T. (2008). Endothelin-1 mediates hypoxia-induced inhibition of voltage-gated K^+^ channel expression in pulmonary arterial myocytes. *American Journal of Physiology-Lung Cellular and Molecular Physiology*.

[88] Huetsch J. C., Walker J., Undem C. (2018). Rho kinase and Na^+^/H^+^ exchanger mediate endothelin-1-induced pulmonary arterial smooth muscle cell proliferation and migration. *Physiological Reports*.

[89] MacLean M. R., Alexander D., Stirrat A. (2000). Contractile responses to human urotensin-II in rat and human pulmonary arteries: effect of endothelial factors and chronic hypoxia in the rat. *British Journal of Pharmacology*.

[90] Eddahibi S., Fabre V., Boni C. (1999). Induction of serotonin transporter by hypoxia in pulmonary vascular smooth muscle cells. Relationship with the mitogenic action of serotonin. *Circulation Research*.

[91] Mair K. M., MacLean M. R., Morecroft I., Dempsie Y., Palmer T. M. (2008). Novel interactions between the 5-HT transporter, 5-HT1B receptors and Rho kinase in vivo and in pulmonary fibroblasts. *British Journal of Pharmacology*.

[92] Maclean M. R., Dempsie Y. (2010). The serotonin hypothesis of pulmonary hypertension revisited. *Advances in Experimental Medicine and Biology*.

[93] MacLean M. R. (2018). The serotonin hypothesis in pulmonary hypertension revisited: targets for novel therapies (2017 Grover Conference Series). *Pulmonary Circulation*.

[94] Oka M., Homma N., Taraseviciene-Stewart L. (2007). Rho kinase-mediated vasoconstriction is important in severe occlusive pulmonary arterial hypertension in rats. *Circulation Research*.

[95] Ward J. P., McMurtry I. F. (2009). Mechanisms of hypoxic pulmonary vasoconstriction and their roles in pulmonary hypertension: new findings for an old problem. *Current Opinion in Pharmacology*.

[96] Firth A. L., Choi I. W., Park W. S. (2012). Animal models of pulmonary hypertension: rho kinase inhibition. *Progress in Biophysics and Molecular Biology*.

[97] Oka M., Fagan K. A., Jones P. L., McMurtry I. F. (2008). Therapeutic potential of RhoA/Rho kinase inhibitors in pulmonary hypertension. *British Journal of Pharmacology*.

[98] Yang X., Lee P. J., Long L., Trembath R. C., Morrell N. W. (2007). BMP4 induces HO-1 via a Smad-independent, p38MAPK-dependent pathway in pulmonary artery myocytes. *American Journal of Respiratory Cell and Molecular Biology*.

[99] Gerthoffer W. T. (2007). Mechanisms of vascular smooth muscle cell migration. *Circulation Research*.

[100] Liu Y., Suzuki Y. J., Day R. M., Fanburg B. L. (2004). Rho kinase-induced nuclear translocation of ERK1/ERK2 in smooth muscle cell mitogenesis caused by serotonin. *Circulation Research*.

[101] Fukumoto Y., Matoba T., Ito A. (2005). Acute vasodilator effects of a Rho-kinase inhibitor, fasudil, in patients with severe pulmonary hypertension. *Heart*.

[102] Ishikura K., Yamada N., Ito M. (2006). Beneficial acute effects of rho-kinase inhibitor in patients with pulmonary arterial hypertension. *Circulation Journal*.

[103] Fagan K. A., Oka M., Bauer N. R. (2004). Attenuation of acute hypoxic pulmonary vasoconstriction and hypoxic pulmonary hypertension in mice by inhibition of Rho-kinase. *American Journal of Physiology-Lung Cellular and Molecular Physiology*.

[104] Abe K., Shimokawa H., Morikawa K. (2004). Long-term treatment with a Rho-kinase inhibitor improves monocrotaline-induced fatal pulmonary hypertension in rats. *Circulation Research*.

[105] Wei H., Zhang D., Liu L., Xia W., Li F. (2019). Rho signaling pathway enhances proliferation of PASMCs by suppressing nuclear translocation of Smad1 in PAH. *Experimental and Therapeutic Medicine*.

[106] Sieber C., Kopf J., Hiepen C., Knaus P. (2009). Recent advances in BMP receptor signaling. *Cytokine & Growth Factor Reviews*.

[107] Davies R. J., Morrell N. W. (2008). Molecular mechanisms of pulmonary arterial hypertension: role of mutations in the bone morphogenetic protein type II receptor. *Chest*.

[108] Du L., Sullivan C. C., Chu D. (2003). Signaling molecules in nonfamilial pulmonary hypertension. *New England Journal of Medicine*.

[109] Takahashi H., Goto N., Kojima Y. (2006). Downregulation of type II bone morphogenetic protein receptor in hypoxic pulmonary hypertension. *American Journal of Physiology-Lung Cellular and Molecular Physiology*.

[110] Lowery J. W., Caestecker M. P. D. (2010). BMP signaling in vascular development and disease. *Cytokine & Growth Factor Reviews*.

[111] Hassoun P. M., Mouthon L., Barbera J. A. (2009). Inflammation, growth factors, and pulmonary vascular remodeling. *Journal of the American College of Cardiology*.

[112] Rabinovitch M., Guignabert C., Humbert M., Nicolls M. R. (2014). Inflammation and immunity in the pathogenesis of pulmonary arterial hypertension. *Circulation Research*.

[113] Shalapour S., Karin M. (2015). Immunity, inflammation, and cancer: an eternal fight between good and evil. *Journal of Clinical Investigation*.

[114] Humbert M., Monti G., Brenot F. (1995). Increased interleukin-1 and interleukin-6 serum concentrations in severe primary pulmonary hypertension. *American Journal of Respiratory and Critical Care Medicine*.

[115] Selimovic N., Bergh C.-H., Andersson B., Sakiniene E., Carlsten H., Rundqvist B. (2009). Growth factors and interleukin-6 across the lung circulation in pulmonary hypertension. *European Respiratory Journal*.

[116] Savale L., Tu L., Rideau D. (2009). Impact of interleukin-6 on hypoxia-induced pulmonary hypertension and lung inflammation in mice. *Respiratory Research*.

[117] Steiner M. K., Syrkina O. L., Kolliputi N., Mark E. J., Hales C. A., Waxman A. B. (2009). Interleukin-6 overexpression induces pulmonary hypertension. *Circulation Research*.

[118] Paulin R., Courboulin A., Meloche J. (2011). Signal transducers and activators of transcription-3/pim1 axis plays a critical role in the pathogenesis of human pulmonary arterial hypertension. *Circulation*.

[119] Bonnet S., Belus A., Hyvelin J.-M., Roux E., Marthan R., Savineau J.-P. (2001). Effect of chronic hypoxia on agonist-induced tone and calcium signaling in rat pulmonary artery. *American Journal of Physiology-Lung Cellular and Molecular Physiology*.

[120] Bonnet S., Archer S. L., Allalunis-Turner J. (2007). A mitochondria-K^+^ channel axis is suppressed in cancer and its normalization promotes apoptosis and inhibits cancer growth. *Cancer Cell*.

[121] Savai R., Al-Tamari H. M., Sedding D. (2014). Pro-proliferative and inflammatory signaling converge on FoxO1 transcription factor in pulmonary hypertension. *Nature Medicine*.

[122] Dansen T. B., Burgering B. M. T. (2008). Unravelling the tumor-suppressive functions of FOXO proteins. *Trends in Cell Biology*.

[123] Laplante M., Sabatini D. M. (2012). mTOR signaling in growth control and disease. *Cell*.

[124] Goncharova E. A. (2013). mTOR and vascular remodeling in lung diseases: current challenges and therapeutic prospects. *The FASEB Journal*.

[125] Goncharov D. A., Kudryashova T. V., Ziai H. (2014). Mammalian target of rapamycin complex 2 (mTORC2) coordinates pulmonary artery smooth muscle cell metabolism, proliferation, and survival in pulmonary arterial hypertension. *Circulation*.

[126] Tang H., Wu K., Wang J. (2018). Pathogenic role of mTORC1 and mTORC2 in pulmonary hypertension. *JACC: Basic to Translational Science*.

[127] Halder G., Johnson R. L. (2011). Hippo signaling: growth control and beyond. *Development*.

[128] Ehmer U., Sage J. (2016). Control of proliferation and cancer growth by the Hippo signaling pathway. *Molecular Cancer Research*.

[129] Zanconato F., Forcato M., Battilana G. (2015). Genome-wide association between YAP/TAZ/TEAD and AP-1 at enhancers drives oncogenic growth. *Nature Cell Biology*.

[130] Bertero T., Oldham W. M., Cottrill K. A. (2016). Vascular stiffness mechanoactivates YAP/TAZ-dependent glutaminolysis to drive pulmonary hypertension. *Journal of Clinical Investigation*.

[131] Bertero T., Cottrill K. A., Lu Y. (2015). Matrix remodeling promotes pulmonary hypertension through feedback mechanoactivation of the YAP/TAZ-miR-130/301 circuit. *Cell Reports*.

[132] Kudryashova T. V., Goncharov D. A., Pena A. (2016). HIPPO-Integrin-linked kinase cross-talk controls self-sustaining proliferation and survival in pulmonary hypertension. *American Journal of Respiratory and Critical Care Medicine*.

[133] Boucherat O., Bonnet S., Paulin R. (2016). The HIPPO-thesis of pulmonary HYPERtension. *American Journal of Respiratory and Critical Care Medicine*.

[134] Kroemer G., Pouyssegur J. (2008). Tumor cell metabolism: cancer’s Achilles’ heel. *Cancer Cell*.

[135] Sutendra G., Michelakis E. D. (2014). The metabolic basis of pulmonary arterial hypertension. *Cell Metabolism*.

[136] Li M., Riddle S., Zhang H. (2016). Metabolic reprogramming regulates the proliferative and inflammatory phenotype of adventitial fibroblasts in pulmonary hypertension through the transcriptional corepressor C-terminal binding protein-1. *Circulation*.

[137] Plecita-Hlavata L., Tauber J., Li M. (2016). Constitutive reprogramming of fibroblast mitochondrial metabolism in pulmonary hypertension. *American Journal of Respiratory Cell and Molecular Biology*.

[138] Dromparis P., Paulin R., Sutendra G., Qi A. C., Bonnet S., Michelakis E. D. (2013). Uncoupling protein 2 deficiency mimics the effects of hypoxia and endoplasmic reticulum stress on mitochondria and triggers pseudohypoxic pulmonary vascular remodeling and pulmonary hypertension. *Circulation Research*.

[139] Sutendra G., Dromparis P., Wright P. (2011). The role of Nogo and the mitochondria-endoplasmic reticulum unit in pulmonary hypertension. *Science Translational Medicine*.

[140] Al-Mehdi A.-B., Pastukh V. M., Swiger B. M. (2012). Perinuclear mitochondrial clustering creates an oxidant-rich nuclear domain required for hypoxia-induced transcription. *Science Signaling*.

[141] Heiden M. G. V., Cantley L. C., Thompson C. B. (2009). Understanding the Warburg effect: the metabolic requirements of cell proliferation. *Science*.

[142] Dai J., Zhou Q., Chen J., Rexius-Hall M. L., Rehman J., Zhou G. (2018). Alpha-enolase regulates the malignant phenotype of pulmonary artery smooth muscle cells via the AMPK-Akt pathway. *Nature Communications*.

[143] Parra V., Bravo-Sagua R., Norambuena-Soto I. (2017). Inhibition of mitochondrial fission prevents hypoxia-induced metabolic shift and cellular proliferation of pulmonary arterial smooth muscle cells. *Biochimica et Biophysica Acta (BBA)-Molecular Basis of Diseas*.

[144] Kovacs L., Cao Y., Han W. (2019). PFKFB3 in smooth muscle promotes vascular remodeling in pulmonary arterial hypertension. *American Journal of Respiratory and Critical Care Medicine*.

[145] Yeligar S. M., Kang B.-Y., Bijli K. M. (2018). PPARgamma regulates mitochondrial structure and function and human pulmonary artery smooth muscle cell proliferation. *American Journal of Respiratory Cell and Molecular Biology*.

[146] Xie X., Li S., Zhu Y. (2018). Egr-1 mediates leptin-induced PPARgamma reduction and proliferation of pulmonary artery smooth muscle cells. *Molecular Biology of the Cell*.

[147] Li H.-H., Hsu H.-H., Chang G.-J. (2018). Prostanoid EP4 agonist L-902, 688 activates PPARgamma and attenuates pulmonary arterial hypertension. *American Journal of Physiology-Lung Cellular and Molecular Physiology*.

[148] Weiss A., Neubauer M. C., Yerabolu D. (2019). Targeting cyclin-dependent kinases for the treatment of pulmonary arterial hypertension. *Nature Communications*.

[149] Wang J., Feng W., Li F. (2019). SphK1/S1P mediates TGF-*β*1-induced proliferation of pulmonary artery smooth muscle cells and its potential mechanisms. *Pulmonary Circulation*.

[150] Sysol J. R., Chen J., Singla S. (2018). Micro-RNA-1 is decreased by hypoxia and contributes to the development of pulmonary vascular remodeling via regulation of sphingosine kinase 1. *American Journal of Physiology-Lung Cellular and Molecular Physiology*.

[151] Luo L., Zheng W., Lian G. (2017). Combination treatment of adipose-derived stem cells and adiponectin attenuates pulmonary arterial hypertension in rats by inhibiting pulmonary arterial smooth muscle cell proliferation and regulating the AMPK/BMP/Smad pathway. *International Journal of Molecular Medicine*.

[152] Xia J., Yang L., Dong L. (2018). Cefminox, a dual agonist of prostacyclin receptor and peroxisome proliferator-activated receptor-gamma identified by virtual screening, has therapeutic efficacy against hypoxia-induced pulmonary hypertension in rats. *Frontiers in Pharmacology*.

